# Associations between maternal physical activity in early and late pregnancy and offspring birth size: remote federated individual level meta‐analysis from eight cohort studies

**DOI:** 10.1111/1471-0528.15476

**Published:** 2018-10-22

**Authors:** S Pastorino, T Bishop, SR Crozier, C Granström, K Kordas, LK Küpers, EC O'Brien, K Polanska, KA Sauder, MH Zafarmand, RC Wilson, C Agyemang, PR Burton, C Cooper, E Corpeleijn, D Dabelea, W Hanke, HM Inskip, FM McAuliffe, SF Olsen, TG Vrijkotte, S Brage, A Kennedy, D O'Gorman, P Scherer, K Wijndaele, NJ Wareham, G Desoye, KK Ong

**Affiliations:** ^1^ MRC Epidemiology Unit University of Cambridge Cambridge UK; ^2^ MRC Lifecourse Epidemiology Unit (University of Southampton) Southampton General Hospital Southampton UK; ^3^ Department of Epidemiology Research Centre for Fetal Programming State Serum Institute Copenhagen Denmark; ^4^ Epidemiology and Environmental Health School of Public Health and Health Professions University at Buffalo Buffalo NY USA; ^5^ Department of Epidemiology University Medical Center Groningen University of Groningen Groningen the Netherlands; ^6^ MRC Integrative Epidemiology Unit School of Social and Community Medicine University of Bristol Bristol UK; ^7^ Obstetrics & Gynaecology UCD Perinatal Research Centre School of Medicine University College Dublin National Maternity Hospital Dublin Ireland; ^8^ Department of Environmental Epidemiology Nofer Institute of Occupational Medicine Lodz Poland; ^9^ Department of Pediatrics University of Colorado School of Medicine Aurora CO USA; ^10^ Department of Public Health Amsterdam Public Health Research Institute, Amsterdam UMC University of Amsterdam Amsterdam the Netherlands; ^11^ Department of Obstetrics & Gynaecology Amsterdam UMC University of Amsterdam Amsterdam the Netherlands; ^12^ Department of Clinical Epidemiology Biostatistics and Bioinformatics Amsterdam Public Health Research Institute Amsterdam UMC University of Amsterdam the Netherlands; ^13^ Institute of Health and Society Newcastle University Newcastle UK; ^14^ NIHR Southampton Biomedical Research Centre University Hospital Southampton NHS Foundation Trust and University of Southampton Southampton UK; ^15^ Department of Epidemiology Colorado School of Public Health University of Colorado Anschutz Medical Campus Denver CO USA; ^16^ 3U Diabetes Consortium and School of Health and Human Performance Dublin City University Dublin Ireland; ^17^ School of Biological Sciences Dublin Institute of Technology Dublin Ireland; ^18^ Department of Obstetrics & Gynaecology Medical University of Graz Graz Austria

**Keywords:** Birth weight, large‐for‐gestational age, macrosomia, physical activity, pregnancy, small‐for‐gestational age

## Abstract

**Objective:**

Evidence on the impact of leisure time physical activity (LTPA) in pregnancy on birth size is inconsistent. We aimed to examine the association between LTPA during early and late pregnancy and newborn anthropometric outcomes.

**Design:**

Individual level meta‐analysis, which reduces heterogeneity across studies.

**Setting:**

A consortium of eight population‐based studies (seven European and one US) comprising 72 694 participants.

**Methods:**

Generalised linear models with consistent inclusion of confounders (gestational age, sex, parity, maternal age, education, ethnicity, BMI, smoking, and alcohol intake) were used to test associations between self‐reported LTPA at either early (8–18 weeks gestation) or late pregnancy (30+ weeks) and the outcomes. Results were pooled using random effects meta‐analyses.

**Main outcome measures:**

Birth weight, large‐for‐gestational age (LGA), macrosomia, small‐for‐gestational age (SGA), % body fat, and ponderal index at birth.

**Results:**

Late, but not early, gestation maternal moderate to vigorous physical activity (MVPA), vigorous activity, and LTPA energy expenditure were modestly inversely associated with BW, LGA, macrosomia, and ponderal index, without heterogeneity (all: *I* ^2^ = 0%). For each extra hour/week of MVPA, RR for LGA and macrosomia were 0.97 (95% CI: 0.96, 0.98) and 0.96 (95% CI: 0.94, 0.98), respectively. Associations were only modestly reduced after additional adjustments for maternal BMI and gestational diabetes. No measure of LTPA was associated with risk for SGA.

**Conclusions:**

Physical activity in late, but not early, pregnancy is consistently associated with modestly lower risk of LGA and macrosomia, but not SGA.

**Tweetable abstract:**

In an individual participant meta‐analysis, late pregnancy moderate to vigorous physical activity modestly reduced birth size outcomes.

## Tweetable abstract

In an individual participant meta‐analysis, late pregnancy moderate to vigorous physical activity modestly reduced birth size outcomes.

## Introduction

The prevalence of childhood obesity has increased worldwide over the last three decades.^1^ Babies born with large‐for‐gestational age (LGA), or with macrosomia [birth weight (BW) above 4000 or 4500 g], have higher risks of obesity and raised metabolic disease markers in childhood compared with babies with appropriate BW.^2^, ^3^ Physical activity during pregnancy is recommended to enhance the health of the mother‐to‐be,^4^ but has also been explored as a potential intervention to lower the risk for LGA and macrosomia.^5^, ^6^, ^7^, ^8^, ^9^, ^10^ Physical activity might be especially appealing if it reduced high BW without increasing the risk of small‐for‐gestational age (SGA) babies. Physical activity during pregnancy might reduce fetal growth by increasing insulin sensitivity and by modulating glucose regulation.^11^, ^12^ Physical activity might also regulate fetoplacental growth by altering the rates of oxygen and nutrient supply.^13^

Recent systematic reviews of randomised controlled trials on the effect of maternal exercise on birth outcomes report modest BW reductions (10–30 g).^14^, ^15^ However, they report wide variation in the types of interventions studied in terms of form, intensity, and volume of exercise. Systematic reviews of observational studies on the association between maternal physical activity during pregnancy with birth size^16^, ^17^ report conflicting results: some studies report an inverse association,^5^, ^6^, ^7^, ^8^, ^9^, ^10^, ^18^, ^19^ some a positive association^20^, ^21^, ^22^ and others no significant association.^23^, ^24^, ^25^, ^26^, ^27^, ^28^ There is also some evidence that the timing of physical activity in pregnancy might be important.^18^, ^29^ The heterogeneity among studies limits the ability to pool published results. One meta‐analysis^17^ reports that ‘high’ physical activity levels were inversely associated with BW, but conversely ‘moderate’ physical activity levels were positively associated with BW. The included studies use different definitions of physical activity level and there is no standardisation with regard to the type and domains of activity or the volume, intensity, and timing. Most studies did not adjust for any confounder.

Here, we examined the association between leisure time physical activity (LTPA) during pregnancy and newborn anthropometric outcomes across a range of prospective cohort studies. Within a consortium created as part of the InterConnect project,^30^ we used a federated meta‐analysis approach,^31^ which allows an individual participant‐level meta‐analysis to be performed remotely. Compared with a literature‐based meta‐analysis, this approach can reduce heterogeneity between studies by allowing harmonisation of exposure and outcome variables, and by allowing the same models to be tested in each study.

## Methods

InterConnect is an EU‐FP7 funded project that optimises the use of existing data by enabling cross‐cohort analyses within consortia without pooling of individual‐level data at a central location. For this research question, eight cohorts with data on physical activity in pregnancy and neonatal outcomes set up a server to allow remote federated analyses and joined the consortium. The collaborative group comprised the following prospective birth cohort studies: the Avon Longitudinal Study of Parents and Children (ALSPAC, UK),^32^, ^33^ the Amsterdam Born Children and their Development study (ABCD, the Netherlands),^34^ the Danish National Birth Cohort (DNBC, Denmark),^35^ the Groningen Expert Center for Kids with Obesity (GECKO)‐Drenthe (the Netherlands),^36^ the Healthy Start Study (HSS, USA),^18^ the Polish Mother and Child Cohort (REPRO_PL, Poland),^37^ the ROLO study (Ireland),^38^ and the Southampton Women's Survey (SWS, UK).^39^ Characteristics of the participating studies are shown in Table S1. Each participating cohort obtained ethical approval from the corresponding local ethics committee (see details at the end). No PPI took place for these analyses.

We included all live‐born singleton full‐term births and excluded mothers with pre‐eclampsia and those with missing information for any of the covariates. The percentage of participants with any missing values across cohorts ranged between 10.2% and 34% for early pregnancy analyses, and between 12.7% and 43.5% for late pregnancy analyses. Funding for this study was received from the European Union Seventh Framework Programme (FP7/2007–2013) under grant agreement no. 602068. Core Outcome Set (COS), and patient involvement (PPI) is not relevant to this study and hence is not described here.

### Physical activity during pregnancy

All studies assessed physical activity during pregnancy by questionnaire. HSS and SWS used interviewer‐administered questionnaires, DNBC used a computer‐assisted telephone interview, and the other studies used self‐administered questionnaires. Table S2 details the questions in each cohort. We harmonised self‐reported data on LTPA during early pregnancy across seven cohorts, and LTPA during late pregnancy across five cohorts. The median gestational age at which mothers replied to questionnaires was 8–18 weeks for early pregnancy, and 30 weeks to 1 day post‐delivery for late pregnancy. LTPA was chosen as it is the domain most amenable to intervention and therefore more relevant for public health recommendations; it was also the most commonly assessed domain across the eight studies. Intensity of reported activities was expressed in metabolic equivalent of energy expenditure (MET) values according to the Compendium of Physical Activity.^40^ Four exposure variables were harmonised: (1) duration of LTPA (hours/week), which included any reported leisure time activity; (2) duration of moderate‐vigorous LTPA (MVPA) (hours/week) including activities with intensity ≥3 MET; (3) duration of vigorous LTPA (VPA) (hours/week) including activities with intensity ≥6 MET; (4) energy expenditure of LTPA (MET‐hours/week) calculated by multiplying duration of LTPA by MET values. Three studies recorded categorical response formats for duration of LTPA (ALSPAC, GECKO, and SWS). These were converted into numerical values, where relevant using the mid‐point of the stated range (i.e. ‘>7 hours/week’ was converted to 7 hours/week; ‘2–6’ to 4; <1 to 0.5; ‘never’ to 0).

### Outcomes

The following outcome variables were harmonised across all studies, based on objective measurements in all studies: BW (g), macrosomia (defined as BW >4000 g), LGA (BW for gestational age >90th percentile according to the INTERGROWTH‐21st Project^41^ and SGA (BW for gestational age <10th percentile according to INTERGROWTH‐21st). Ponderal Index, a measure of leanness (corpulence) [weight/length^3^ (kg/m^3^)] at birth was harmonised for six cohorts. Percent (%) body fat at birth was available for three cohorts. Of these, one (HSS) assessed newborn body fat using air displacement plethysmography (PEAPOD), while skinfold thickness measurements were available in HSS, SWS, and in a subset of ROLO (*n* = 219). Triceps and subscapular skinfolds were used to estimate % body fat using the algorithm reported by Slaughter et al.^42^ % body fat = 1.21 × (triceps skinfold + subscapular skinfold) −0.008 × (triceps skinfold + subscapular skinfold)^2^ − 1.7.

### Potential modifiers

The following potential modifying variables were harmonised across the studies: infant sex, maternal obesity (BMI: ≤20 kg/m^2^, >20–30 kg/m^2^, >30 kg/m^2^), maternal ethnicity (white, black, other), and gestational diabetes mellitus (GD: yes, no). Maternal weight was objectively measured in five cohorts and self‐reported in three cohorts at varying times in early pregnancy up to week 18 of gestation. We applied a uniform correction factor to weights measured later than 12 weeks gestation derived by weight gain curves based on repeated maternal weight measures in the ALSPAC study. There was wide variation in definitions of ethnicity across cohorts; the ‘other ethnicity’ category includes a variety of Asian, Hispanic, and other ethnic groups. GDM was defined using biochemical data at weeks 24–28 in HSS and ROLO, and by a combination of medical records and self‐reports in the other studies.

### Potential confounders and other covariates

Potential confounders were not harmonised because, in federated analysis models involving random‐effects meta‐analysis of the arising study‐specific estimates, this would not impact the summary effect estimates and *P*‐values. However, confounder variables were reasonably comparable across studies. Smoking in pregnancy was a dichotomous variable (yes/no) in all studies except DNBC, which determined the number of cigarettes/week. Alcohol intake was considered as units of alcohol/week in ALSPAC, DNBC, and SWS; glasses/week in ABCD; and as categorical variables in GECKO (none, <1 glass/week, 1–6 glasses/week, 7+ glasses/week), HSS (none, once per month or less, twice per month or more), REPRO_PL (yes/no), and ROLO (yes/no). Educational attainment was considered as a categorical variable in most cohorts (range 2–6 levels) except ABCD, which recorded ‘years of education after elementary school’. Parity (number of previous live births) was self‐reported in all studies, and maternal age at delivery was calculated from mother's date of birth and delivery date.

### Statistical analyses

All analyses were conducted using R within the DataSHIELD federated meta‐analysis library.^43^ In this process, individual participant data from contributing studies are held securely on servers at each study location.^30^ A computer within the network sends analytical commands that request each local server to undertake an analysis locally and return non‐identifiable summary statistics. The result of this process is mathematically equivalent to an individual participant meta‐analysis with the advantage that data remain within the governance structure of each single cohort study.^30^

To analyse data, we used generalised linear models in each study. Each model was fitted in a federated manner using the iterative reweighted least squares process.^31^ The primary models included MVPA duration as exposure and each outcome (BW, macrosomia, LGA, SGA, ponderal index, % body fat) separately. Moderate to vigorous activity was chosen as the primary exposure because it has higher validity than lower intensity activities^44^ also, the majority of existing guidelines recommend moderate intensity physical activity for pregnant women.^45^ The adjusted models included each exposure separately (LTPA duration, MVPA duration, VPA duration, LTPA energy expenditure) with each outcome (BW, macrosomia, LGA, SGA, ponderal index, % body fat), and were adjusted for gestational age (except for LGA and SGA), infant sex, parity, maternal age, smoking, alcohol, maternal education, and ethnicity. Further models were additionally adjusted for maternal early pregnancy BMI. A schematic diagram of the analysis plan is shown in Figure S1. All covariates were chosen a priori based on literature evidence. To explore which covariate contributed most to heterogeneity, we conducted further analyses by including each potential confounding variable one at a time. Physical activity is likely to exert its effect on birth size by altering maternal metabolic pathways such as glucose metabolism, and there is evidence of its association with GDM.^46^ Therefore, GDM was added in a subsequent model to explore its possible mediating effect. We explored the possible modifying effect of infant sex, maternal obesity, maternal ethnicity, and GDM by including interaction terms in the model. These potential effect modifiers were chosen a priori. The levels of physical activity and their effects on health differ across ethnic groups.^47^ In pregnant women, both obesity and GDM might alter physiological characteristics that affect their ability to exercise.^48^ All models were conducted separately for early and late pregnancy physical activity. Early pregnancy physical activity measures were available for ALSPAC, ABCDS, DNBC, HSS, REPRO‐PL, ROLO, and SWS. Late pregnancy physical activity measures were available for DNBC, GECKO, HSS, REPRO_PL, and SWS. Regression analyses were conducted for each individual study, and then random‐effects meta‐analysis was used to combine the effect estimates. A random effects approach was chosen owing to the reported heterogeneity between other published studies. Heterogeneity was assessed using the *I* ^2^ statistic.

## Results

For early pregnancy physical activity analyses, 72 694 participants from seven studies were included (57 807 across six studies for ponderal index; 3039 in three studies for % body fat). For late pregnancy analyses, the available sample was 58 820 from five studies (57 172 across four studies for ponderal index; 2792 in two studies for % body fat). Maternal and infant characteristics are presented in Table 1. Mean BW ranged between 3356 and 4135 g for male infants, and between 3217 and 3963 g for female infants. ROLO infants had the highest mean BW and highest prevalence of macrosomia (51.8%) and LGA (61.7%), reflecting their inclusion of only secundigravid women whose first baby had been macrosomic. Among the other cohorts, macrosomia prevalence ranged between 5.6% in HSS and 21.7% in DNBC, and LGA between 8.7% in HSS and 30.2% in GECKO. SGA prevalence ranged between 0.8% in ROLO and 9.4% in HSS. Median ponderal index at birth ranged between 20.2 in REPRO_PL and 27.8 in SWS, and body fat was 10, 11, and 16% in HSS, SWS, and ROLO, respectively.

**Table 1:** Study population characteristics

	ALSPAC	ABCD	DNBC	GECKO	HSS	REPRO_PL	ROLO	SWS
a **, median (IQR)**	26.2 (24.7–27.8)		24.9 (23.5–26.5)		26.9 (24.9–29.2)	20.2 (18.9‐ 21.6)	27.1 (25.3–29.3)	27.8 (26.3–29.2)
**% Body fat** b **, median (IQR)**					10 (8–12)		16 (14–18)	11 (10–13)
**Early pregnancy physical activity, median (IQR)**								
LTPA (hours/week)	4.0 (0.5–5.5)	2.0 (0.5–4.3)	0.0 (0.0–1.0)		3.0 (1.0–5.8)	4.0 (0.0–7.0)	1.7 (1.0–2.3)	6.5 (3.2–11.5)
MVPA (hours/week)	4.0 (0.5–5.0)	1.5 (0.0–3.5)	0.0 (0.0–1.0)		1.5 (0.0–3.5)	0.0 (0.0–0.0)	0.3 (0.0–1.0)	1.2 (0.3–3.0)
LTPA EE (Met‐hours/week)	15.2 (3.0–25.2)	8.1 (1.7–19.3)	0.0 (0.0–6.0)		10.2 (3.1–23.6)	16.5 (0.0–33.0)	4.5 (2.0–7.8)	17.5 (8.7–32.1)
Women doing vigorous PA, *n* (%)	604 (6.6)	1876 (29)	4321 (8.0)		244 (23.1)	84 (8.5)	61 (9.8)	810 (42.5)
**Late pregnancy physical activity, median (IQR)**								
LTPA (hours/week)			0.0 (0.0–1.0)	1.0 (1.0–1.0)	2.0 (0.5–3.6)	5.0 (0.0–8.0)		7.0 (3.4–12.0)
MVPA (hours/week)			0.0 (0.0–1.0)	0.3 (0.0–1.0)	0.0 (0.0–1.5)	0.0 (0.0–0.0)		0.8 (0.1–2.3)
Women doing vigorous PA, *n* (%)			1599 (2.9)		61 (5.7)	77 (8.3)		443 (24.1)
LTPA EE (Met‐hours/week)			0.0 (0.0–3.0)	1.0 (0.0–4.0)	6.3 (1.5–11.9)	19.8 (0.0–33.0)		16.7 (8.5–31.1)
**Maternal age** (y), mean (SD)	28.5 (4.7)	30.9 (5.1)	30.1 (4.2)	30.8 (4.2)	28 (6.1)	29.0 (4.2)	32.2 (4.1)	30.6 (3.7)
**Maternal BMI**								
Mean (SD)	22.5 (4.3)	24.0 (4.1)	24.7 (4.1)	24.7 (4.7)	26.7 (6.0)	22.8 (3.6)	26.6 (4.8)	26 (4.8)
Overweight, *n* (%)	1257 (13.8)	1447 (22.3)	14 896 (27.7)	320 (24)	334 (31.6)	162 (16.4)	233 (37.7)	606 (32)
Obese, *n* (%)	586 (6.4)	527 (8.1)	5546 (10.3)	169 (12.6)	225 (21.3)	39 (3.9)	114 (18.4)	323 (17)
**GDM,** ***n*** **(%)**	41 (0.4)	76 (1.1)	380 (10.7)	44 (3.2)	43 (4)	34 (3.4)	12 (2)	19 (1)
**Ethnicity**								
White	8867 (98)	4490 (69.4)	53 671 (100)	1321 (99)	814 (76.5)	982 (100)	612 (97.5)	1840 (96.8)
Black	77 (0.8)	486 (7.6)	0 (0)	0 (0)	162 (15.3)	0 (0)	2 (0.3)	10 (0.5)
Other	114 (1.2)	1488 (23)	0 (0)	14 (1)	78 (7.2)	0 (0)	14 (2.2)	52 (2.7)

EE, energy expenditure; GDM, gestational diabetes mellitus; LGA, large for gestational age; LTPA, leisure‐time physical activity; MVPA, moderate to vigorous leisure time physical activity; SGA, small for gestational age.

a Sample size available for late pregnancy physical activity analyses were: DNBC = 53 684, HSS = 1044, REPRO_PL = 919, SWS = 1838.

Sample size available for analyses of Ponderal index for early pregnancy analyses were: ALSPAC = 7118, DNBC = 53 487, HSS = 976, REPRO_PL = 977, ROLO = 523, SWS = 1844; for late pregnancy analyses: DNBC = 53 500, HSS = 968, REPRO_PL = 915, SWS = 1789.

b Sample size available for analyses of % body fat for early pregnancy analyses were: HSS = 988, ROLO = 189, SWS = 1862; for late pregnancy analyses: HSS = 987, SWS = 1805.

Reported levels of maternal LTPA during pregnancy varied across studies, with DNBC women having the lowest levels in both periods (64% of women reporting no LTPA). Among the other cohorts, median LTPA duration ranged from 2.0 to 6.5 hours/week for early pregnancy, and 1–7 hours/week for late pregnancy. Median MVPA levels ranged from 0 to 4 hours/week for early pregnancy, and 0–0.8 hours/week for late pregnancy. The proportion of women reporting any MVPA decreased from the early pregnancy in the four studies with data at both time points (DNBC, 34%; HSS, 72%; REPRO_PL, 20%; SWS, 84%) to late pregnancy (DNBC, 25%; HSS, 49%; REPRO_PL, 12%; SWS, 78%). The proportion of women reporting any VPA was low in most cohorts (range: 6.6–42.5%) and decreased in late pregnancy (range: 2.9–24.1%).

### Physical activity associations in early pregnancy

Early pregnancy maternal LTPA was not associated with any measure of offspring birth size (Tables 2, [Link], [Link]). Heterogeneity across studies was high in unadjusted models (*I* ^2^ = 79–86% for BW, macrosomia, and LGA, Table S1), but was substantially reduced after adjustments for potential confounders (0–54%, Table 2). In sensitivity models, with stepwise inclusion of covariates, ethnicity and maternal education contributed the most to (positive) confounding in some individual studies, with non‐white ethnicity being associated with both lower BW and lower LTPA, and maternal education being associated with both higher BW and higher LTPA (not shown).

**Table 2:** Associations between physical activity during pregnancy and offspring birth size

	BW (grams)	Macrosomia	LGA	Ponderal index	SGA
	RR, 95% CI *I* ^2^	RR, 95% CI *I* ^2^	RR, 95% CI *I* ^2^	Beta, 95% CI *I* ^2^	Beta, 95% CI *I* ^2^
**Physical activity** **Early pregnancy**					
LTPA (hours/week)	−0.86 (−2.33, 0.61) 23%	0.99 (0.98, 1,01) 51%	0.99 (0.98, 1,00) 46%	0.0 (−0.01, 0.01) 0%	0.99 (0.98, 1.01) 0%
MVPA (hours/week)	−1.38 (−3.77, 1.01) 41%	1.00 (0.98, 1,01) 52%	1.00 (0.98, 1,01) 43%	0.00 (−0.01, 0.01) 0%	0.99 (0.98, 1.00) 0%
VPA (hours/week)	−1.38 (−3.77, 1.01) 41%	1.00 (0.98, 1,01) 52%	1.00 (0.98, 1,01) 43%	0.00 (−0.05, 0.04) 18%	0.99 (0.98, 1.00) 0%
LTPAEE (met‐hours/week)	−0.14 (−0.58, 0.30) 49%	1.00 (0.99, 1,00) 53%	0.99 (0.99, 1,00) 38%)	0.00 (0.00, 0.00) 0%	0.99 (0.99, 1.00) 0%
Physical activity Late pregnancy					
LTPA (hours/week)	−2.22 (−5.54, 1.0) 64%	**0.98 (0.96, 1.00)** **37%**	**0.98 (0.97, 0.99)** **0%**	**−0.01 (−0.02, 0.00)** **13%**	0.99 (0.97, 1.01) 0%
MVPA (hours/week)	**−6.43 (−9.12, −3.74)** **0%**	**0.96 (0.94, 0.98)** **0%**	**0.97 (0.96, 0.98)** **0%**	**−0.02 (−0.03, 0.00)** **0%**	1.01 (0.97, 1.03) 0%
VPA (hours/week)	**−22.0 (−31.3, −12.7)** **0%**	**0.89 (0.84, 0.95)** **0%**	**0.89 (0.84, 0.94)** **0%**	**−0.07 (−0.13, −0.02)** **0%**	1.06 (0.96, 1.17) 0%
LTPAEE (met‐hours/week)	**−0.93 (−1.43, −0.42)** **9%**	**0.99 (0.99, 0.99)** **0%**	**0.99 (0.99, 0.99)** **0%**	**0.00 (−0.01, 0.00)** **0%**	0.99 (0.99, 1.00) 0%

EE, energy expenditure; LGA, large for gestational age; LTPA, leisure time physical activity; MVPA, moderate to vigorous leisure time activity; SGA, small for gestational age; VPA, vigorous leisure time activity.

Models are adjusted for gestational age, sex, parity, maternal age, smoking, alcohol, maternal education, and ethnicity. Statistically significant associations are highlighted in bold.

### Physical activity associations in late pregnancy

**Figure 1:**
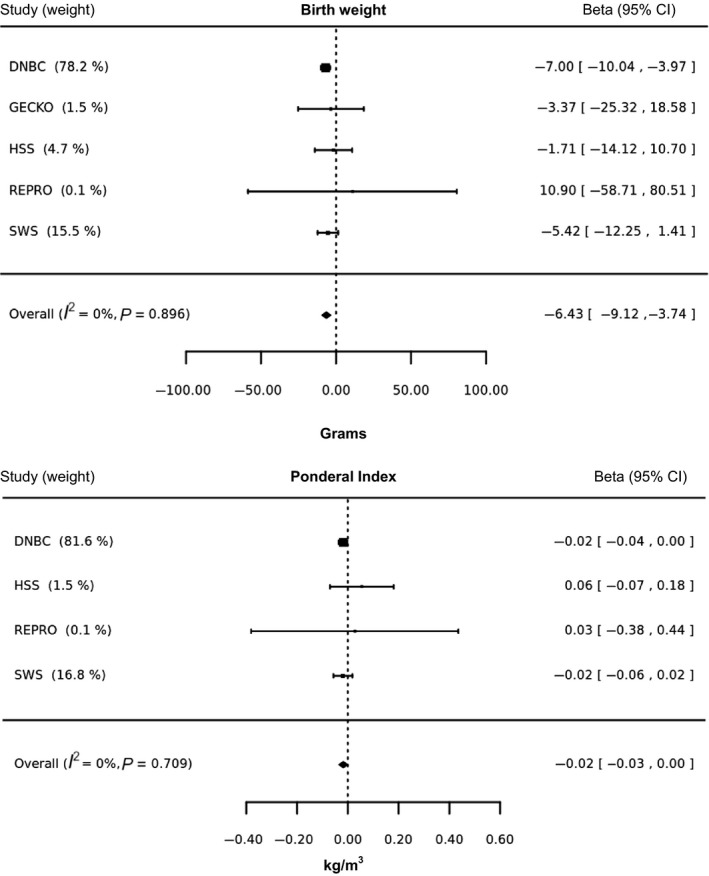
Forest plots for late pregnancy moderate to vigorous activity (hours/week) associated with birth weight and ponderal index. Associations were adjusted for gestational age, sex, parity, maternal age, smoking, alcohol, maternal education, and ethnicity. *n* = 58 820 except for ponderal index (*n* = 57 172).

**Figure 2:**
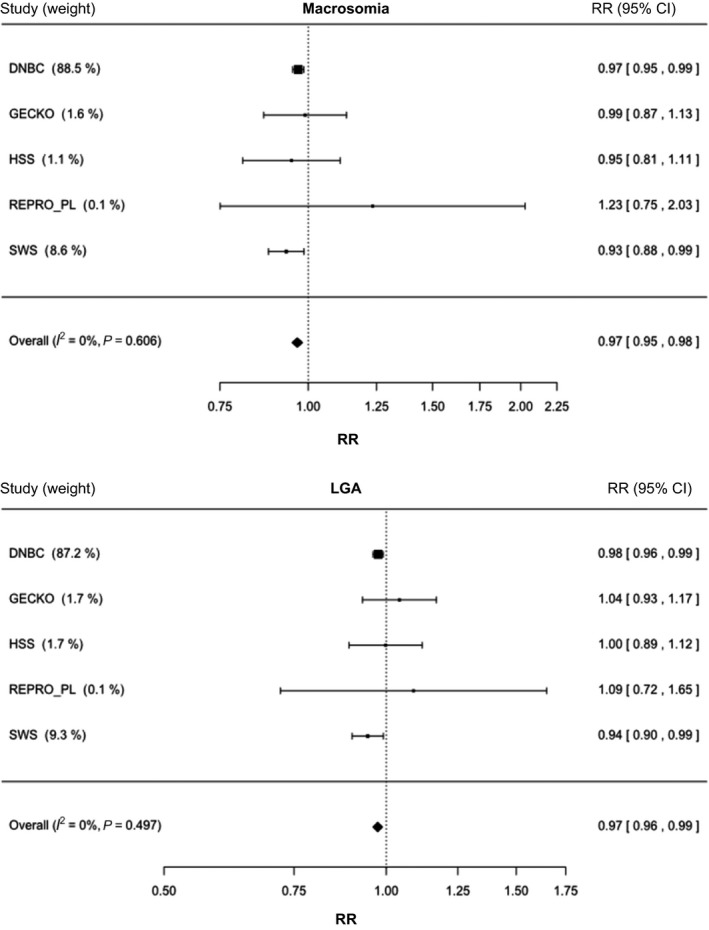
Forest plots for late pregnancy moderate to vigorous activity (hours/week) associated with relative risk of macrosomia and large for gestational age (LGA). Associations were adjusted for gestational age, sex, parity, maternal age, smoking, alcohol, maternal education, and ethnicity. *n* = 58 820.

Late pregnancy maternal MVPA (Figures 1 and 2, Table 2), VPA, and LTPA energy expenditure (Tables 2 andS3) were inversely associated with all birth size outcomes (except for % body fat and SGA) in adjusted models. For each +1 hour/week of MVPA, offspring BW was lower by 6.4 g (95% CI: 9.1, 3.7; *P* <0.001) and ponderal index by 0.02 kg/m^3^ (95% CI: 0.03, 0.00; *P* = 0.02); the relative risks of macrosomia and LGA were lower by 4% (95% CI: 2, 6; *P* <0.001) and 3% (95% CI: 2, 4; *P* <0.01), respectively. No association was found for SGA (OR: 0.99, 95% CI: 0.98, 1.00) and % body fat (−0.01, 95% CI: −0.04, 0.02). VPA showed larger associations with BW (−22 g/hour/week; 95% CI: −31.3, −12.7; *P* <0.001), ponderal index (−0.07 units; 95% CI: −0.13, −0.02; *P* <0.01), macrosomia (lower by 11%, 95% CI: 5, 16; *P* <0.01) and LGA (lower by 11%, 95% CI: 5, 16 *P* <0.001), and no association with % body fat (−0.05; 95% CI: −0.17, −0.06) and SGA (OR: 1.01, 95% CI: 0.96, 1.16). The associations with late pregnancy LTPA were not mediated by GDM and persisted after further adjustment for early pregnancy maternal BMI (Table S5).

No interaction with ethnicity, infant sex, GDM, or maternal obesity was found in either pregnancy period for LTPA and birth size (all *P*‐values for interactions >0.05).

## Discussion

### Main findings

In this large cross‐cohort analysis of up to 72 694 individuals, we found small but consistent inverse associations between maternal LTPA during late, but not early, pregnancy and offspring birth size. Each additional hour/week of MVPA in late pregnancy was associated with 6.4 g lower birth weight and 4% and 3% relative reductions in risk of macrosomia and LGA, respectively, without increasing the risk of SGA.

### Strengths and limitations

A major strength of our approach was the planned individual level analysis across several cohort studies. Compared with the inconsistent findings of published literature‐based systematic reviews, heterogeneity between study estimates was substantially reduced by consistent confounding adjustment and by harmonisation of exposures and outcomes. The remote federated analysis approach avoided the need to physically pool individual‐level data, and hence substantially reduced the governance burdens and associated time delays, and avoided barriers due to limitations of consent and research ethics permissions. Another strength is that we were able to analyse the differential association of timing and intensity of physical activity in pregnancy with offspring birth size outcomes.

However, there were some limitations in our approach. Physical activity was self‐reported in all included studies, and only a few of the questionnaires were validated. Physical activity questionnaires are susceptible to measurement error related to both recall and social desirability with validity estimated between 0.25 and 0.4.^49^ However, they are able to rank individuals according to activity levels.^50^ Furthermore, validity is higher among women than men and for vigorous intensity compared with lighter intensity activities.^44^ It remains a challenge to identify thresholds of physical activity in terms of health benefits. Contributing studies used different questionnaires with varying ways of assessing LTPA, which made harmonisation challenging. For example, some listed specific activities (e.g. ‘swimming’, ‘walking’) while others asked only about categories of activities (i.e. ‘moderate, ‘vigorous’), which included some activities outside of leisure time. Intensity information was not available in all questionnaires, which meant assumptions had to be made when assigning MET values. Differences in average LTPA levels across the studies might therefore reflect differences in methods or real population differences. The timing of questionnaire administration differed across studies, particularly for early pregnancy LTPA. Unfortunately, data were unavailable on clinical outcomes associated with LGA and macrosomia (such as shoulder dystocia, 3rd or 4th degree laceration), or on pregnancies not resulting in live birth. Future analyses including such outcomes would be highly informative. Our use of international INTERGROWTH‐21st Project data to define LGA and SGA led to unequal numbers for those outcomes and limited the statistical power to detect a possible association between VPA and SGA. Although we adjusted for many confounding factors, residual confounding cannot be ruled out. Limited geographical and ethnic diversity restricted the power to detect modifying factors. One participating study (DNBC) was substantially larger than the other studies and accounted for more than 70% of the sample size in the analyses. Whilst the dominance of this study in driving results should be acknowledged, it is noteworthy that, in adjusted models, heterogeneity was reduced from >70 to 0% in most analyses, thus highlighting the consistency across studies and the generalisability of results.

### Interpretations

The direction of our associations is consistent with some previous individual studies;^5^, ^6^, ^7^, ^8^, ^9^, ^10^, ^18^, ^19^ however, other studies reported null^23^, ^24^, ^25^, ^26^, ^27^, ^28^ or even directionally opposite results.^20^, ^21^, ^22^ A recent meta‐analysis^17^ reports that a ‘moderate’ level of physical activity was positively associated with BW, while a ‘high’ level of physical activity was inversely associated with BW. However, those results were based on a mixture of adjusted and unadjusted models, and their reported meta‐analysis of only the adjusted models showed null associations for both moderate and high levels of physical activity. Furthermore, in that meta‐analysis, there was substantial heterogeneity, with *I* ^2^ values >80%. We demonstrate here that more consistent adjustment for confounding reduced heterogeneity between individual study estimates from *I* ^2^ >70% to 0% in several analyses. Furthermore, adjustment for ethnicity and maternal education avoided spurious positive associations between early pregnancy physical activity and birth size. We harmonised the intensity of activities by assigning the same MET values for similar reported activities across studies. Although the diverse nature of the questionnaires used in the individual studies made harmonisation challenging, MVPA was less heterogeneous than other activity variables, particularly in late pregnancy; this may be because our harmonised MVPA variable was more robust to underlying methodological differences across studies.

The timing of PA associations with LTPA during late, but not early, pregnancy is also consistent with some reported studies^18^, ^29^, ^51^ Clapp et al.^51^ reported inverse associations with newborn adiposity or BW only for late pregnancy physical activity. Hopkins and Cutfield^29^ conjectured that high volume exercise only in the first half of pregnancy increased BW, but if performed throughout pregnancy or only in the second half of pregnancy, it reduced BW. They suggested that the timing of physical activity caused different fetoplacental adaptations.

Regarding intensity of LTPA, we found that late pregnancy MVPA, VPA, and energy expenditure, but not duration of LTPA, were inversely associated with offspring birth size. Some previous studies have assessed the impact of physical activity intensity on offspring birth size, with some findings consistent with ours,^21^, ^22^, ^51^ but others reported null results.^27^, ^52^, ^53^, ^54^ Different adjustment factors and different definitions, timing, and categories of physical activities might lead to inconsistent findings between studies. Although the proportion of women reporting any VPA was small, our results suggest that changes in birth size outcomes are dependent on the intensity of LTPA, with larger effects observed with higher intensity. It is possible that LTPA intensity needs to reach a certain threshold before it has an effect on nutrient supply to the fetus. Alternatively, higher intensity recreational activities may be easier to recall and less prone to measurement error.^44^

Our observed associations remained significant after adjustment for maternal BMI, possibly suggesting that the effect of physical activity on birth size is only partially mediated by maternal weight; however, we did not have measures of late pregnancy maternal weight gain and BMI. Independent of maternal weight, physical activity increases maternal insulin sensitivity,^12^, ^55^ reduces maternal glucose, and hence might reduce glucose transfer to the fetus.^56^ These metabolic changes are more marked at higher intensities and volumes of exercise and in late pregnancy.^11^, ^29^

## Conclusion

In conclusion, LTPA energy expenditure, MVPA, and VPA during late, but not early, pregnancy had a small but significant and consistent inverse association with offspring birth size. Larger effects were observed with higher intensity of physical activity. Compared with the inconsistent findings of reviews of published reports, this remote federated individual‐level analysis substantially reduced heterogeneity between individual studies by allowing consistent adjustment for confounding and careful harmonisation of exposures and outcomes.

## Disclosure of interests

None declared. Completed disclosure of interests form available to view online as supporting information.

## Contributions to authorship

GD, KKO, SP contributed to planning the study. SP, KW, SB, AK, DOG, and KKO coordinated harmonisation of all variables. TB and PS conducted the federated remote statistical analyses. SP, KKO, DOG, and SB interpreted the results. SP wrote the article. SP, TB, SB, KW, NJW, GD, KKO, SRC, CG, KK, LKK, EOB, KP, KAS, MHZ, BW, CA, PRB, CC, EC, DD, WH, HMI, FM, SFO, and TGV contributed to the analysis plan, the production of the paper, the harmonisation algorithms, and the review of the manuscript.

## Details of ethics approval

Avon Longitudinal Study of Parents and Children (ALSPAC): ethical approval for the study was obtained from ALSPAC Ethics and Law Committee and the Local Research Ethics Committees. Amsterdam Born Children and their Development study (ABCD): approval of the study was obtained from the Central Committee on Research Involving Human Subjects in The Netherlands, the medical ethics review committees of the participating hospitals, and the Registration Committee of the Municipality of Amsterdam. Danish National Birth Cohort (DNBC, Denmark): approved by the Committee on Biomedical Research Ethics under case number (KF) 01‐471/94. Groningen Expert Center for Kids with Obesity (GECKO )‐Drenthe: approved by the Medical Ethics Committee of the University Medical Center Groningen (UMCG). Healthy Start Study (HSS): approved by the Colorado Multiple Institutional Review Board. Polish Mother and Child Cohort (REPRO_PL): approved by the Ethical Committee of the Nofer Institute of Occupational Medicine, Łódź, Poland (Decision No. 7/2007). ROLO study: approved by the Ethics Committee at the National Maternity Hospital, June 2006. Southampton Women's Survey (SWS): approved by South Central—Hampshire B Research Ethics Committee.

## Funding

InterConnect: the research leading to these results received funding from the European Union Seventh Framework Programme (FP7/2007–2013) under grant agreement no. 602068. Amsterdam Born Children and their Development study (ABCD): the ABCD study was supported by grants from the Netherlands Organization for Health Research and Development (ZonMW) and The Netherlands Heart Foundation. Genotyping was funded by the BBMRI‐NL grant CP2013‐50. M.H. Zafarmand was supported by BBMRI‐NL (CP2013‐50). T.G.M. Vrijkotte was supported by ZonMW (TOP 40–00812–98–11010). Avon Longitudinal Study of Parents and Children (ALSPAC): we are grateful to all the families who took part in this study, the midwives for their help in recruiting them, and the Avon Longitudinal Study of Parents and Children (ALSPAC) team, including interviewers, computer and laboratory technicians, clerical workers, research scientists, volunteers, managers, receptionists, and nurses. The UK Medical Research Council and Wellcome (grant ref: 102215/2/13/2) and the University of Bristol provide core support for ALSPAC. Danish National Birth Cohort (DNBC, Denmark): the Danish National Research Foundation has established the Danish Epidemiology of Science Centre that initiated and created the DNBC. The cohort is furthermore a result of a major grant from this foundation. Additional support for the DNBC is obtained from the Pharmacy Foundation, the Egmont Foundation, the March of Dimes Birth Defect Foundation, the Augustinus Foundation, and the Health Foundation. Groningen Expert Center for Kids with Obesity (GECKO)‐Drenthe: the GECKO Drenthe birth cohort was funded by an unrestricted grant of Hutchison Whampoa Ltd, Hong Kong, and supported by the University of Groningen, Well Baby Clinic Foundation Icare, Noordlease, and Youth Health Care Drenthe. Healthy Start Study (HSS) was funded by the following NIH funding sources: R01DK076645, UL1TR00108. Polish Mother and Child Cohort (REPRO_PL) is supported in part by funds from National Centre for Research and Development, Poland (grant no. PBZ‐MEiN‐/8/2/2006; contract no. K140/P01/2007/1.3.1.1) and grant PNRF‐218‐AI‐1/07 from Norway through the Norwegian Financial Mechanism within the Polish–Norwegian Research Fund. ROLO study was funded by the Health Research Board of Ireland, with additional financial support from the National Maternity Hospital Medical Fund. Southampton Women's Survey (SWS) was supported by grants from the Medical Research Council, National Institute for Health Research Southampton Biomedical Research Centre, University of Southampton and University Hospital Southampton National Health Service Foundation Trust, and the European Union's Seventh Framework Programme (FP7/2007‐2013), project EarlyNutrition (grant 289346). SB, KW, NW and KO are supported by the Medical Research Council (Unit Programme numbers: MC_UU_12015/1, MC_UU_12015/2 and MC_UU_12015/3).

## Supporting information


**Figure S1.** Schematic representation of the analysis plan.

**Table S1.** Characteristics of the eight contributing cohort studies.

**Table S2.** Questions asked in the participating cohorts used for harmonisation of leisure time physical activity exposure.

**Table S3.** Unadjusted^a^ associations between physical activity during pregnancy and offspring birth size.

**Table S4.** Associations between physical activity during pregnancy and % body fat.

**Table S5.** Associations between late pregnancy physical activity and offspring birth size with additional adjustments for maternal early pregnancy BMI and GDM.
