# Supplementary figures and images for: Associations between maternal physical activity in early and late pregnancy and offspring birth size: remote federated individual level meta‐analysis from eight cohort studies
Source: BJOG. 2018 Oct 22;126(4):459–70. doi: 10.1111/1471-0528.15476 (PMC6330060; doi:10.1111/1471-0528.15476)

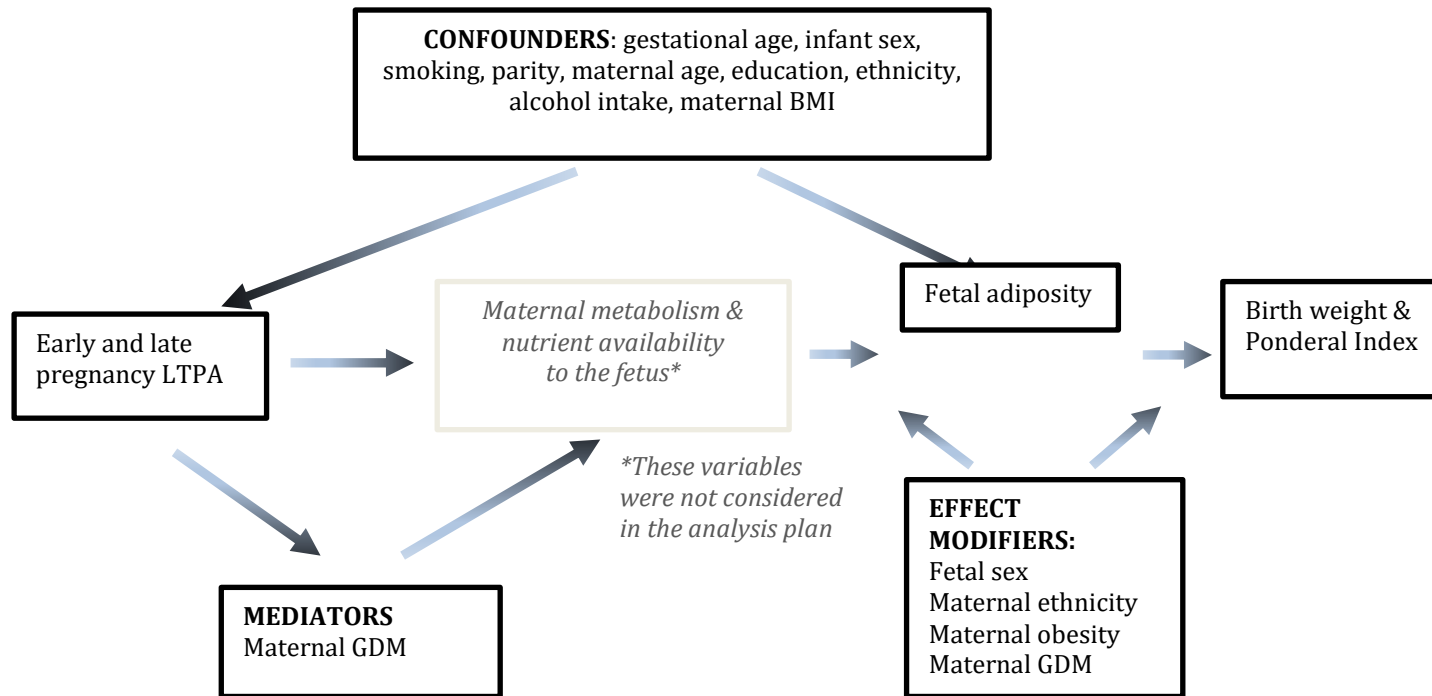

**Figure S1.** Schematic representation of the analysis plan

Supplement: Supplementary file 1 — Figure S1. Schematic representation of the analysis plan. [file BJO-126-459-s001.pdf]
