# Supplementary material for: Associations between maternal physical activity in early and late pregnancy and offspring birth size: remote federated individual level meta‐analysis from eight cohort studies
Source: BJOG. 2018 Oct 22;126(4):459–70. doi: 10.1111/1471-0528.15476 (PMC6330060; doi:10.1111/1471-0528.15476)
Supplement: Supplementary file 2 — Table S1. Characteristics of the eight contributing cohort studies. [file BJO-126-459-s002.pdf]

Table S1. Characteristics of the eight contributing cohort studies

Study name	Study design	Original inclusion/exclusion criteria	Location	Recruitment time-frame	Sample size, Early pregnancy	Sample size, Late pregnancy
ALSPAC[a] [32, 33]	Population based cohort	Pregnant women resident in Avon	Avon, UK	1990-1992	9,058	
ABCD [34]	Population based cohort	Pregnant women resident in Amsterdam (first prenatal visit)	Amsterdam, Netherlands	2003-2004	6,464	
DNBC [35]	Population based cohort	Pregnant women resident in Denmark (first GP visit)	Denmark	1996-2002	53,671	53,684
GECKO [36]	Population based cohort	Pregnant women resident in Drenthe province (third trimester)	Drenthe, Netherlands	2006-2007		1,335
HSS [18]	Population based cohort	Pregnant women expecting single birth, no previous stillbirth or prior diabetes	Colorado, US	2010-2014	1,054	1,044
REPRO_PL [37]	Population based cohort	Pregnant women expecting single birth, no serious chronic disease or assisted reproduction	Poland	2007-2011	982	919
ROLO [38]	Randomised controlled trial	Secundigravid women whose first baby was macrosomic (birth weight >4kg)	Dublin, Ireland	2007-2011	617	
SWS [39]	Population based cohort	Non-pregnant women resident in Southampton	Southampton, UK	1998-2002	1,902	1,838

ALSPAC= Avon Longitudinal Study of Parents and Children; ABCD= Amsterdam Born Children and their Development study; DNBC= Danish National Birth Cohort; GECKO= Groningen Expert Center for Kids with Obesity; HSS= Healthy Start Study; REPRO-PL= Polish Mother and Child Cohort; ROLO=; SWS=Southampton Women's Survey

[a]ALSPAC fully searchable data dictionary is available at: http://www.bristol.ac.uk/alspac/researchers/access/
