# Supplementary material for: Associations between maternal physical activity in early and late pregnancy and offspring birth size: remote federated individual level meta‐analysis from eight cohort studies
Source: BJOG. 2018 Oct 22;126(4):459–70. doi: 10.1111/1471-0528.15476 (PMC6330060; doi:10.1111/1471-0528.15476)
Supplement: Supplementary file 3 — Table S2. Questions asked in the participating cohorts used for harmonisation of leisure time physical activity exposure. [file BJO-126-459-s003.pdf]

Table S2. Questions asked in the participating cohorts used for harmonisation of leisure time physical activity exposure

	When	Question	Frequency/duration
**ALSPAC**	18 w and 32 w	**1. How much do you do the following at present?**<br>1. Jogging 2 Aerobic 3. Ante-natal exercises 4. Keep fit exercises 5.Yoga 6.Squash 7. Tennis/badminton 8.Swimming 9.Brisk walking 10.Weight training 11.Cycling 12.Other exercises	>7h/w, 2-6h/w, <1h/w, never
**ABCD**	15.6 w (median)	**In your spare time did you:**<br>1. Did you take walks for fun in the past week? 2. Did you ride a bicycle in the past week? 3. Did you play sports in the past week? (for example: tennis, handball, gymnastics, fitness, skating, and swimming) 4. Did you do any other physical exercise in your spare time in the past week, for example, working in the garden and doing odd jobs around the house (do not include household activities).<br>For each question: At what pace do you usually do this?<br>• relaxed pace • average pace • brisk pace	mins/week
**DNBC**	12 w and 30w	**Do you get any kind of exercise? What kind of exercise?**<br>1 special gymnastics/aerobics for pregnant women 2 aerobics/gymnastics 3 dancing 4 cycling 5 fast walk 6 jogging, orienteering 7 ball games 8 swimming 9 fitness, health centres 10 badminton 11 tennis 12 horse back riding 13 other___________________	times/week; mins/time
**GECKO**	3rd trimester	**Do you perform at this moment physical activity at moderate intensity for half an hour per day?**	No, not weekly, Yes, namely ____ times per week (fill in number).
**HSS**	17 w, 27 w, 1d post-delivery (median)	**For fun and exercise how much time did you:**<br>1.Walking slowly 2.Walking more quickly 3.Walking quickly up hills 4. Jogging 5.Prenatal exercise class 6. Swimming 7.Dancing 8. Other	Days/w and hour/day
**REPRO_PL**	8-12w, 20-24w, 30-34w	**In current pregnancy are you physically active** (for example are you doing gymnastics, cycling, swimming, walking)<br>**Type of physical activity** (please write all forms)	hours/week
**ROLO**	first antenatal visit	**How many times/w on average do you do for more than 20 minutes:**<br>1) STRENUOUS EXERCISE (HEART BEATS RAPIDLY) (e.g. running, jogging, hurling, camogie, football, soccer, squash, basketball, judo, roller skating, vigorous swimming, vigorous long distance cycling, advanced aerobics)<br>2) MODERATE EXERCISE (NOT EXHAUSTING) (e.g. fast walking, tennis, badminton, easy swimming, easy cycling, popular and folk dancing, intermediate aerobics, heavy gardening)<br>3) MILD EXERCISE (MINIMAL EFFORT) (e.g. yoga, golf, easy walking, fishing from river bank, bowling, beginners aerobics, archery, light gardening)	Times/w (x20mins)
**SWS**	11w 34w	**During the past three months, how often have you done**<br>1. Strenuous exercise which normally makes your heart beat rapidly AND leaves you breathless e.g. jogging, vigorous swimming or cycling, aerobics.<br>2. Moderate exercise which normally leaves you exhausted but not breathless, e.g. brisk walking, dancing, easy swimming or cycling, badminton, sailing.<br>3. Gentle exercise which normally leaves you tired but not exhausted, e.g. walking, heavy housework (including washing windows and polishing), gardening, DIY, golf.	None, once every 2-3m, once a month, once a fortnight, 1-2times/w, 3-6times/w, once/day, >once/day. Hours/mins of each session

ALSPAC= Avon Longitudinal Study of Parents and Children; ABCD= Amsterdam Born Children and their Development study; DNBC= Danish National Birth Cohort; GECKO= Groningen Expert Center for Kids with Obesity; HSS= Healthy Start Study; REPRO-PL= Polish Mother and Child Cohort; ROLO=; SWS=Southampton Women' Survey
