# Supplementary material for: Associations between maternal physical activity in early and late pregnancy and offspring birth size: remote federated individual level meta‐analysis from eight cohort studies
Source: BJOG. 2018 Oct 22;126(4):459–70. doi: 10.1111/1471-0528.15476 (PMC6330060; doi:10.1111/1471-0528.15476)
Supplement: Supplementary file 4 — Table S3. Unadjusteda associations between physical activity during pregnancy and offspring birth size. [file BJO-126-459-s004.pdf]

Table S3. Unadjusted[a] associations between physical activity during pregnancy and offspring birth size

	BW (grams)	Macrosomia	LGA	Ponderal Index	SGA
**Physical activity Early pregnancy**	*Beta, 95% CI* *I^2*	*RR, 95% CI* *I^2*	*RR, 95% CI* *I^2*	*Beta, 95% CI* *I^2*	*Beta, 95% CI* *I^2*
LTPA (h/w)	0.30 (-3.39, 3.99) 86%	0.99 (0.97, 1.02) 82%	0.99 (0.97, 1.01) 80%	0.00 (-0.02, 0.02) 71%	0.98 (0.96, 1.00) 41%
MVPA (h/w)	-0.18 (-5.46, 5.09) 86%	1.00 (0.97 1.03) 82%	0.99 (0.97, 1.02) 81%	-0.01 (-0.02, 0.01) 37%	0.98 (0.95, 1.01) 47%
VPA (h/w)	-1.62 (-12.53, 9.28) 74%	0.97 (0.91 1.04) 61%	0.98 (0.92, 1.03) 67%	-0.01 (-0.07, 0.04) 30%	1.01 (0.96, 1.06) 0%
LTPAEE (met-h/w)	0.17 (-0.72, 1.06) 87%	1.00 (0.99 1.00) 82%	1.00 (0.99, 1.00) 79%	0.00 (0.00, 0.00) 65%	0.99 (0.99, 1.00) 61%
**Physical activity Late pregnancy**					
LTPA (h/w)	-2.85 (-9.21, 3.52) 90%	0.98 (0.94, 1.01) 81%	0.98 (0.95, 1.00) 78%	-0.01 (-0.04, 0.01) 84%	0.99 (0.97, 1.01) 0%
MVPA (h/w)	**-9.53 (-12.84, -6.23) 6%**	**0.94 (0.93, 0.96) 0%**	**0.95 (0.94, 0.97) 0%**	**-0.03 (-0.05, -0.01) 15%**	1.01 (0.98, 1.04) 0%
VPA (h/w)	**-24.1 (-33.7, -14.6) 0%**	**0.88 (0.83, 0.94) 0%**	**0.88 (0.83, 0.93) 0%**	**-0.0i9 (-0.15, -0.04) 0%**	1.06 (0.96, 1.17) 0%
LTPAEE (met-h/w)	-0.92 (-2.04, 0.19) 73%	**0.99 (0.98, 0.99) 0%**	**0.99 (0.99, 0.99) 7%**	**0.00 (-0.01, 0.00) 68%**	0.99 (0.99, 1.00) 0%

[a]Unadjusted models include only gestational age and sex;
LGA= Large for gestational age; SGA= Small for gestational age; LGA= Large for gestational age; SGA= Small for gestational age; LTPA=leisure time physical activity; MVPA=moderate to vigorous leisure time activity; EE=energy expenditure. VPA= vigorous leisure time activity. Statistically significant associations are highlighted in bold.
