# Supplementary material for: Associations between maternal physical activity in early and late pregnancy and offspring birth size: remote federated individual level meta‐analysis from eight cohort studies
Source: BJOG. 2018 Oct 22;126(4):459–70. doi: 10.1111/1471-0528.15476 (PMC6330060; doi:10.1111/1471-0528.15476)
Supplement: Supplementary file 5 — Table S4. Associations between physical activity during pregnancy and % body fat. [file BJO-126-459-s005.pdf]

Table S4. Associations between physical activity during pregnancy and % body fat

	Unadjusted [a]	Adjusted [b]
	Beta, 95% CI I^2	*Beta, 95% CI* I^2
**Physical activity Early pregnancy (N=3,039)**		
LTPA (h/w)	-0.03 (-0.10, 0.04) 66%	-0.01 (-0.03, 0.02) 27%
MVPA (h/w)	-0.01 (-0.07, 0.04) 70%	-0.02 (-0.04, 0.02) 18%
VPA (h/w)	-0.09 (-0.30, 0.12) 80	-0.06 (-0.25, 0.12) 75%
LTPAEE (met-h/w)	0.00 (-0.02, 0.01) 80%	0.00 (-0.01, 0.00) 46%
**Physical activity Late pregnancy (N=2,792)**		
LTPA (h/w)	-0.01 (-0.01, 0.02) 0%	0.00 (-0.01, 0.01) 0%
MVPA (h/w)	-0.01 (-0.04, 0.03) 0%	-0.01 (-0.04, 0.02) 0%
VPA (h/w)	-0.06 (-0.18, 0.06) 0%	-0.05 (-0.17, 0.06) 0%
LTPAEE (met-h/w)	0.00 (0.00, 0.01) 80%	0.00 (-0.01, 0.00) 0%

[a] Unadjusted models include gestational age and sex; [b] adjusted models include gestational age, sex, parity, maternal age, smoking, alcohol, maternal education and ethnicity. LTPA=leisure time physical activity; MVPA=moderate to vigorous leisure time activity; EE=energy expenditure. VPA= vigorous leisure time activity. Statistically significant associations are highlighted in bold.
