# Supplementary material for: Associations between maternal physical activity in early and late pregnancy and offspring birth size: remote federated individual level meta‐analysis from eight cohort studies
Source: BJOG. 2018 Oct 22;126(4):459–70. doi: 10.1111/1471-0528.15476 (PMC6330060; doi:10.1111/1471-0528.15476)
Supplement: Supplementary file 6 — Table S5. Associations between late pregnancy physical activity and offspring birth size with additional adjustments for maternal early pregnancy BMI and GDM. [file BJO-126-459-s006.pdf]

Table S5. Associations between late pregnancy physical activity and offspring birth size with additional adjustments for maternal early pregnancy BMI and GDM

	**Additionally adjusted for maternal BMI**			
	**BW (grams)**	**Macrosomia**	**LGA**	**Ponderal Index**
	Beta, 95% CI<br>*I^2*	*RR, 95% CI*<br>*I^2*	*RR, 95% CI*<br>*I^2*	*Beta, 95% CI*<br>*I^2*
**Physical activity**<br>**Early pregnancy**				
LTPA (h/w)	-1.98 (-3.91, -0.06)<br>13%	0.98 (0.97, 0.99)<br>0%	0.98 (0.97, 0.99)<br>0%	-0.01 (-0.03, 0.0)<br>0%
MVPA (h/w)	-4.61 (-7.26, -1.96)<br>0%	0.97 (0.95, 0.99)<br>0%	0.98 (0.96, 0.99)<br>1%	-0.01 (-0.02, 0.00)<br>0%
VPA (h/w)	-16.28 (-25.48, -7.08)<br>0%	0.92 (0.86, 0.98)<br>0%	0.91 (0.86, 0.96)<br>0%	-0.05 (-0.11, 0.00)<br>0%
LTPAEE (met-h/w)	-0.67 (-1.11, -0.22)<br>0%	0.99 (0.99, 0.99)<br>0%	0.99 (0.99, 1.00)<br>0%	0.00 (-0.01, 0.00)<br>0%
	**Additionally adjusted for maternal GDM**			
	**BW (grams)**	**Macrosomia**	**LGA**	**Ponderal Index**
	Beta, 95% CI<br>*I^2*	*RR, 95% CI*<br>*I^2*	*RR, 95% CI*<br>*I^2*	*Beta, 95% CI*<br>*I^2*
**Physical activity**<br>**Late pregnancy**				
LTPA (h/w)	-2.11 (-5.46, 1.4)<br>64%	0.98 (0.96, 1.00)<br>38%	0.98 (0.97, 0.99)<br>0%	-0.01 (-0.02, 0.00)<br>13%
MVPA (h/w)	-6.32 (-9.00, -3.64)<br>0%	0.96 (0.94, 0.98)<br>0%	0.97 (0.96, 0.98)<br>0%	-0.02 (-0.03, 0.00)<br>0%
VPA (h/w)	-21.61 (-30.92, -12.30)<br>0%	0.90 (0.84, 0.95)<br>0%	0.89 (0.84, 0.94)<br>0%	-0.07 (-0.12, -0.02)<br>0%
LTPAEE (met-h/w)	-0.89 (-1.41, -0.37)<br>11%	0.99 (0.99, 0.99)<br>0%	0.99 (0.99, 0.99)<br>0%	0.00 (-0.01, 0.00)<br>0%

Adjusted for gestational age, sex, parity, maternal age, smoking, alcohol, maternal education, ethnicity.

BW= birth weight; LGA= Large for gestational age; LTPA=leisure time physical activity; MVPA=moderate to vigorous leisure time physical activity; EE=energy expenditure; VPA= vigorous leisure time activity
